# Effects of simulated climate change conditions of increased temperature and [CO_2_] on the early growth and physiology of the tropical tree crop, *Theobroma cacao* L.

**DOI:** 10.1093/treephys/tpad116

**Published:** 2023-09-11

**Authors:** Julián Fernando Mateus-Rodríguez, Fiona Lahive, Paul Hadley, Andrew J Daymond

**Affiliations:** Centro de Investigación Palmira, Corporación Colombiana de Investigación Agropecuaria – AGROSAVIA, Intersección Carrera 36A con Calle 23, Palmira, Valle del Cauca, Postcode 753533, Colombia; School of Agriculture, Policy and Development, University of Reading, Earley Gate, Reading RG6 6EU, UK; School of Agriculture, Policy and Development, University of Reading, Earley Gate, Reading RG6 6EU, UK; School of Agriculture, Policy and Development, University of Reading, Earley Gate, Reading RG6 6EU, UK

**Keywords:** abiotic stress, cacao, carbon dioxide, leaf gas exchange, vapour pressure deficit

## Abstract

Despite multiple studies of the impact of climate change on temperate tree species, experiments on tropical and economically important tree crops, such as cacao (*Theobroma cacao* L.), are still limited. Here, we investigated the combined effects of increased temperature and atmospheric carbon dioxide concentration ([CO_2_]) on the growth, photosynthesis and development of juvenile plants of two contrasting cacao genotypes: SCA 6 and PA 107. The factorial growth chamber experiment combined two [CO_2_] treatments (410 and 700 p.p.m.) and three day/night temperature regimes (control: 31/22 °C, control + 2.5 °C: 33.5/24.5 °C and control + 5.0 °C: 36/27 °C) at a constant vapour pressure deficit (VPD) of 0.9 kPa. At elevated [CO_2_], the final dry weight and the total and individual leaf areas increased in both genotypes, while the duration for individual leaf expansion declined in PA 107. For both genotypes, elevated [CO_2_] also improved light-saturated net photosynthesis (*P*_n_) and intrinsic water-use efficiency (*i*WUE), whereas leaf transpiration (*E*) and stomatal conductance (*g*_s_) decreased. Under a constant low VPD, increasing temperatures above 31/22 °C enhanced the rates of *P*_n_, *E* and *g*_s_ in both genotypes, suggesting that photosynthesis responds positively to higher temperatures than previously reported for cacao. However, dry weight and the total and individual leaf areas declined with increases in temperature, which was more evident in SCA 6 than PA 107, suggesting the latter genotype was more tolerant to elevated temperature. Our results suggest that the combined effect of elevated [CO_2_] and temperature is likely to improve the early growth of high temperature-tolerant genotypes, while elevated [CO_2_] appeared to ameliorate the negative effects of increased temperatures on growth parameters of more sensitive material. The evident genotypic variation observed in this study demonstrates the scope to select and breed cacao varieties capable of adapting to future climate change scenarios.

## Introduction

Cacao (*Theobroma cacao* L.) is an important commodity crop for the production of chocolate, cosmetics, beverages and other derivative products (Lima et al. 2011). An estimated 4,818,000 tonnes of cacao beans were produced in 2021/2022, mainly by smallholder farmers in tropical regions of Africa, Asia and America (ICCO 2023). As with all crops, cacao has the potential to be negatively affected by climate change. The concentration of atmospheric carbon dioxide ([CO_2_]) has been increasing since the beginning of the Industrial Revolution, and under the Shared Socioeconomic Pathways 3–7.0, this has been predicted to reach the region of 700 p.p.m. by 2080 (IPCC 2021). Cumulative emissions of CO_2_ and other greenhouses gases are resulting in an increase in global temperatures, leading to changes in weather patterns. Both [CO_2_] and air temperature are important climatic factors affecting plant growth and development (Ainsworth and Long 2005, van der Kooi et al. 2016). In C_3_ plants, elevated [CO_2_] generally has positive effects on plant growth (expressed as greater dry matter accumulation) as a result of enhanced photosynthesis and improved plant water status due to partial stomata closure as well as greater light-use efficiency (Conroy et al. 1990, Drake et al. 1997, Ainsworth and Long 2005, Leakey et al. 2009). Like in other tropical woody species, temperature plays a critical role in cacao growth and development (De Almeida and Valle 2007, Lahive et al. 2019). Balasimha et al. (1991) reported that the optimal temperature for photosynthesis in cacao plants grown under field conditions ranged from 31 to 33 °C, above which photosynthesis declined. Temperature increases above optimum can negatively affect plant growth and accelerate the development rate, potentially reducing final productivity (Hatfield and Prueger 2015). Other impacts of high temperatures include lower photosynthetic efficiency due to reduced Rubisco activity, increased photorespiration and stomatal closure due to higher vapour pressure deficit (VPD) (Krause et al. 2015, Slot and Winter 2016). Thus, physiological and developmental responses of cacao plants to changes in temperature vary according to whether they are subjected to temperatures above or below the optimal range (Raja Harun and Hardwick 1988, Hadley et al. 1994, Hebbar et al. 2020). For example, shoot growth rate and the number of leaf flushes were higher at a day temperature of 30 °C compared with cacao plants grown at a cooler temperature of 23.3 °C (Sale 1969). A temperature of 30 °C also increased the leaf number and leaf area (Sale 1968). Similarly, when comparing the growth responses to temperature regimes of three cacao-growing areas (Bahia, Brazil; Tafo, Ghana; Lower Perak, Malaysia) simulated in a greenhouse experiment, the highest growth rate was observed under the Malaysian (warmest) conditions of 32.5/22.5 °C maximum/minimum (Daymond and Hadley 2004). Furthermore, some genotypes appeared to be more responsive to temperature changes than others. Studying the effects of temperature and VPD on cacao, Raja Harun and Hardwick (1988) reported that temperatures ranging from 20 to 30 °C did not markedly affect photosynthesis, but at temperatures above 30 °C, photosynthesis decreased; however, the authors suggested that this response was an indirect effect of VPD-induced stomatal closure limiting carbon uptake. Recently, Hebbar et al. (2020), Mensah et al. (2022) and Ríos-Bolívar et al. (2022) have also shown that increasing temperature above 30 °C reduced the photosynthesis and growth of cacao plants. However, VPD was again not controlled in these studies, which is likely to have influenced the photosynthetic responses. Research is needed to understand the direct temperature response in cacao independent of VPD and to determine the potential optimum temperature for physiological performance.

In cacao, positive effects of elevated [CO_2_] on young cacao plants have been demonstrated (Baligar et al. 2005, 2008, 2021*a*, 2021*b*, Lahive et al. 2018). Cacao seedlings grown at elevated [CO_2_] (~700 p.p.m.) exhibited enhanced mineral nutrient uptake and increased shoot and root growth compared with plants grown at ambient [CO_2_] (380 p.p.m.) (Baligar et al. 2005). Baligar et al. (2008) reported a 33% increase in photosynthesis with [CO_2_] raised from 85 to 680 p.p.m., without significant changes above 680 p.p.m.. The authors also noted that elevated [CO_2_] led to a 65% decrease in stomatal conductance (*g*_s_). Increased intrinsic water-use efficiency (iWUE) at elevated [CO_2_] in cacao has been observed as a consequence of enhanced photosynthesis rather than decreases in *g*_s_ (Lahive et al. 2018). Recently, Baligar et al. (2021*a*) also reported differences between genotypes in dry weight, root length, height, leaf area, specific leaf area (SLA), relative growth rate, net assimilation rate and nutrient uptake among seven young cacao genotypes grown in elevated [CO_2_].

Under non-limiting water conditions, elevated [CO_2_] may significantly mitigate negative effects of warming, particularly in some C_3_ crops (Lee 2011). For example, DaMatta et al. (2018) reported that improved photosynthetic functioning under [CO_2_] enrichment limited high temperature-induced reductions in photosynthesis in *Coffea arabica* and *Coffea canephora*. However, Kumari et al. (2019) demonstrated that, depending on the cultivar, the improvement in growth and yield at elevated [CO_2_] can be counteracted by high temperatures in pea (*Pisum sativum*). Similarly, Zuidema et al. (2020) working with the sub-tropical forest species *Toona ciliata* reported that the effects of elevated [CO_2_] on tropical tree growth could be less stimulatory at warmer temperatures than commonly expected. Despite the economic importance of cacao, there is little information on the combined effects of increased temperature and [CO_2_] on cacao physiology. Here, under conditions of constant high humidity (controlled at kPa), we explore how elevated temperature and [CO_2_] affect the growth and physiology of two contrasting cacao genotypes. We tested the hypotheses that: (i) growth parameters and leaf-level photosynthetic traits (*P*_n_, *E*, *g*_s_ and *i*WUE) are negatively affected by predicted temperature increases of +2.5 and +5.0 °C above the current average temperature where cacao is cultivated in West Africa (Max/Min of 31/22 °C); (ii) growth parameters and leaf-level photosynthetic traits are enhanced at elevated [CO_2_]; and (iii) elevated [CO_2_] can ameliorate the possible negative effects of high temperature on growth parameters and leaf-level photosynthetic traits of cacao. The responses of two genotypes belonging to two different genetic groups were compared in the present study.

## Materials and methods

### Plant material

Two contrasting juvenile cacao genotypes: SCA 6, from the genetic cluster Contamana, and PA 107, from the genetic cluster Marañon (Motamayor et al. 2008), were used as the basis of the study. Seeds of PA 107 were provided by the Cocoa Research Institute of Ghana (CRIG) and were raised in 1-L pots containing a mixture of sand, gravel and vermiculite (1:2:2 v:v:v) from 11 June to 2 October 2018 in the International Cocoa Quarantine Centre (ICQC, R) greenhouses at the University of Reading. The plants were maintained under tropical conditions (day/night temperature regime of 25–32/20 °C, respectively) at ambient [CO_2_] and were irrigated six times daily with a modified Long Ashton nutrient solution for cacao (End 1990). The nutrient solution contained per litre of water: potassium nitrate (KNO_3_; 0.43 g), ammonium nitrate (NH_4_NO_3_; 0.39 mL of 18%w/w), potassium sulphate (K_2_SO_4_; 0.120 g), magnesium sulphate (MgSO_4_; 0.24 g), potassium dihydrogen phosphate (KH_2_PO_4_; 0.15 g), iron as EDTA (0.03 g) and boric acid (H_3_BO_3_; 0.01 g), manganese sulphate (MnSO_4_; 0.001 g), zinc sulphate (ZnSO_4_; 0.02 g), ammonium molybdate ((NH_4_)_6_Mo_7_O_24_; 0.001 g) and copper sulphate (CuSO_4_; 0.001 g). The pH was maintained at 5.7 through injection of acid into the nutrient mixing tank. The acid stock tank contained nitric acid (2.5 L) and phosphoric acid (H_3_PO_4_) (1.25 L) mixed into 80 L of water.

On 3 October 2018, the plants were transferred to a temperature-controlled glasshouse at the Crops and Environment Laboratory, University of Reading (51°26′15.89″ N, 0°, 56′2.73″W), for acclimatization and were transplanted into 5-L pots filled with the same substrate and were subjected to the same irrigation regime. The environmental conditions in the glasshouse were set to a day/night temperature regime of 32/19 °C. Supplementary lighting (using 400 W high pressure sodium lamps) was used to extend the day length to 12 h and to increase ambient light levels; shade screens were used when light levels exceeded 648 μmol m^−2^ s^−1^. The SCA 6 plants that were produced through in vitro propagation using the somatic embryogenesis method (Guillou et al. 2018) were provided by Nestlé Research Centre in Tours, France. These plants were transferred to the UK on 25 July 2018 and were maintained in the same temperature-controlled glasshouse at the Crops and Environment Laboratory at the University of Reading. On 15 August 2018, plants were transplanted into 5-L pots filled with the same sand, gravel and vermiculite substrate; irrigation regimes and environmental conditions were maintained as described for the PA 107 plants.

### Experimental conditions and treatments

**Figure 1:**
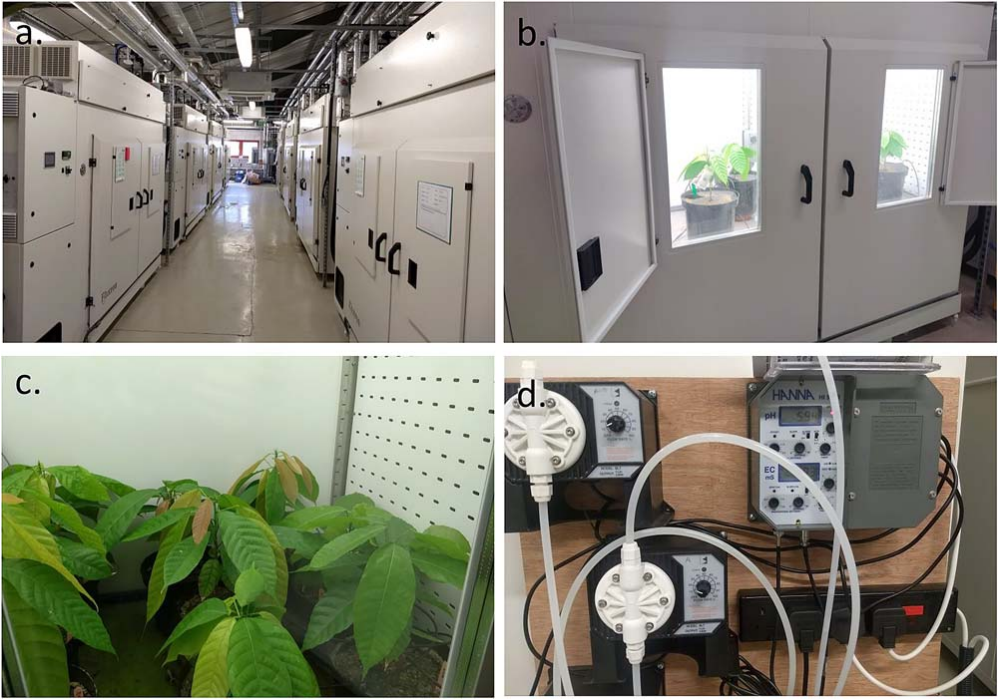
Growth cabinets used for the experiment (a and b), juvenile cacao plants (c) and fertigation control (d).

The experiment started on 10 October 2018 and continued for 88 days. Plants of SCA 6 and PA 107 of similar height and stem diameter were transferred into 12 growth cabinets, each with a growth area of 1.5 m^2^ and 2000 L growth volume (model HGC 1514; Weiss Gallenkamp, UK) (Figure 1a–c) in the Crops and Environment Laboratory, University of Reading. Temperature, relative humidity, lighting and [CO_2_] were monitored using SpecView SCADA control software (SpecView Ltd, East Sussex, UK).

Nine plants per genotype were placed randomly in each half of the cabinet (see Figure S1 available as Supplementary data at *Tree Physiology* Online) and were repositioned fortnightly within each cabinet throughout the experiment to minimize the environmental variation associated with specific positions within the cabinet. An automatic drip system (Figure 1d) irrigated the plants four times per day (06:00, 11:00, 15:00 and 18:00 h) for 5 min at each irrigation (each pot received 2/3 L per day) by using the same modified Long Ashton nutrient solution as used in the glasshouses. Each cabinet was set to provide a 12-h photoperiod through high frequency fluorescent lamps (Master TL5 HO 54 W/840 cool white, Philips Lighting, Amsterdam, The Netherlands) with light intensity at the upper canopy level maintained at an average of 450 μmol m^−2^ s^−1^ PAR. The PAR at canopy height was measured regularly with a portable light meter (SKR 100, Skye Instruments Ltd, Llandrindod Wells, UK). The plant growth rate differed between treatment; so, as the plants grew taller, the canopy height in each cabinet was adjusted by lowering the shelves to maintain a constant distance between the top of the plants and the light source and therefore a similar light intensity across the cabinets.

The experimental design comprised of three temperature and two [CO_2_] treatments in a factorial design (6 treatment combinations in total) as follows: [CO_2_]: ambient (target of 410 p.p.m.) and elevated (target of 700 p.p.m.), day/night temperature: T1 (31/22 °C, control), T2 (33.5/24.5 °C, control + 2.5 °C) and T3 (36/27 °C, control + 5.0 °C), the control simulating the average diurnal temperature regime across the cacao-growing region in Ghana (data obtained from the Ghana Meteorological Service). Each treatment combination was replicated in two different cabinets (see Figure S1 available as Supplementary data at *Tree Physiology* Online), and the plants were considered as replicates within each cabinet. The temperature regimes were set to follow a daily sine wave temperature profile; the maximum and minimum temperatures were maintained from 13:00 to 15:00 h and from 03:00 to 07:00 h, respectively. A constant VPD of 0.9 kPa was maintained across the temperature treatments to avoid the confounding effect of varying evaporative demand between temperature treatments (Balasimha et al. 1991). Environmental variables in the growth cabinets are summarized in Table 1. The [CO_2_] achieved for the elevated treatment were very close to the target but was slightly higher than the target in the ambient treatment (Table 1).

**Table 1:** Average temperatures (°C), CO_2_ concentration (p.p.m.) and VPD (kPa) logged throughout the 88 days of the experimental period.

Treatments		Temperature (°C)	[CO_2_] (p.p.m.)	VPD (kPa)
Temp (°C)	[CO_2_]			
31/22	Ambient	25.9 (±0.1)	460.5 (±1.8)	0.93 (±0.005)
	Elevated	25.9 (±0.1)	696.7 (±1.9)	0.91 (±0.001)
33.5/24.5	Ambient	28.4 (±0.1)	434.3 (±2.2)	0.91 (±0.001)
	Elevated	28.4 (±0.1)	701.1 (±2.7)	0.92 (±0.001)
36/27	Ambient	30.9 (±0.1)	486.4 (±1.7)	0.91 (±0.001)
	Elevated	30.9 (±0.1)	699.4 (±4.2)	0.91 (±0.001)

### Leaf gas exchange measurements

Instantaneous net photosynthesis rate (*P*_n_, *μ*mol m^−2^ s^−1^), transpiration rate (*E*, mmol m^−2^ s^−1^) and stomatal conductance (*g*_s_, mol m^−2^ s^−1^) were measured on the youngest fully expanded and hardened leaf from three plants per genotype in each cabinet using a portable infrared gas analyser fitted with an artificial light attachment and an internal CO_2_ source (LCpro-SD, ADC BioScientific, Great Amwell, Herts, UK). Measurements were performed on Days 27, 55 and 72 between 09:00 and 13:00 h at 696 μmol PAR m^−2^ s^−1^, which can be considered as saturating for cacao (Baligar et al. 2008, Lahive et al. 2018); [CO_2_] was set to the growth concentration (i.e., ∼410 and 700 p.p.m. for ambient and elevated CO_2_ treatments, respectively), and the temperature was set to correspond to the maximum temperature treatments of the cabinets being measured (either 31, 33.5 and 36 °C). The VPD was maintained at ~0.9 kPa across the range of temperatures, and the flow rate in the cuvette was set to 200 μmol s^−1^. Intrinsic water-use efficiency (*i*WUE*, μ*mol mol^−1^) was calculated as the ratio of *P*_n_ to *g*_s_.

### Chlorophyll fluorescence parameters

The maximum quantum efficiency of photosystem II (measured as *F*_v_*/F*_m_ ratio) and the performance index (PI) were measured using a Handy PEA chlorophyll fluorimeter (Hansatech Instruments Ltd, Norfolk, UK) on the same leaves as used for gas exchange measurements. The leaves were dark-adapted using specialized clips for at least 30 min before the measurements were made.

### Measurements of leaf traits

Leaf length (cm) and chlorophyll content (μg cm^−2^) were measured twice weekly for the first 30 and 46 days of development, respectively, on the first newly emerged leaf of a flush, on three plants per genotype in each cabinet. Leaf length was recorded using a measuring tape. Chlorophyll content was measured using a CL-01 portable chlorophyll meter (Hansatech Instruments Ltd). The readings were converted to chlorophyll content (μg cm^−2^) using the linear regression for cacao: *c* = (1.945 × chlorophyll meter reading) + 11.392), as reported by Daymond et al. (2011).

Flushing interval and the number of expanded leaves per flush were recorded three times per week on three plants per genotype in each cabinet. Flushing interval was measured as the number of days between the unrolling of the last leaf of a flush and the unrolling of the first leaf of the subsequent flush (Lahive 2015). Stomatal density (SD, stomata mm ^−2^) was determined before the last destructive harvest. Leaf epidermal imprints were taken from the abaxial surface using clear nail varnish and adhesive cellophane tape on three plants per genotype per cabinet. Three images per imprint were examined, and digital images obtained using a Leitz Dialux 20 light microscope with a Leica DFC450 digital camera attached by using Leica Application suite version 4.6.2 (Leica Microsystems, Wetzlar, Germany). ImageJ version 2.2 analysis software (Rueden et al. 2017) was used for image processing and to count stomata per unit area at 400× magnification.

### Dry weight determinations

Destructive harvests were performed at the beginning (*n =* 3 per genotype) and at the end of the experiment (Day 88; *n* = 6 per genotype per treatment combination). Plants harvested at the beginning were representative of plants going into the experiment. At each harvest, the plants were cut at the base of the stem; the total leaf number and fresh weight (g) of roots (after washing to remove residues from the substrate), stems and petioles and leaves were recorded. Dry weights (g) were recorded after the samples were dried to a constant weight in a ventilated drying oven at 70 °C for at least 48 h. Above- and below-ground dry weight allocation was calculated as a percentage of total plant dry weight. The leaf area (cm^2^) of fresh samples was measured using a WD3 WinDIAS leaf image analysis system (Delta-T Devices Ltd, Cambridge, UK). The specific leaf area (SLA) (cm^2^ g^−1^) was calculated as the ratio of total leaf area to total leaf dry weight. Dried subsamples of leaves were ground to a fine powder for laboratory determinations of leaf carbon and nitrogen concentration using a LECO CNH628 Series Elemental Analyser (LECO Corporation, MI, USA).

### Statistical analysis

All analyses and figure preparation were carried out using the open-source statistical software R, version 4.0.4 (R Core Team 2021). The experiment was considered to be a completely randomized, split plot design with three factors, with the combination of [CO_2_] and temperature (growth cabinets) as the main plots and genotypes as sub-plots. Before statistical analyses, the data were first checked for normality and homoscedasticity by Shapiro–Wilk and Levene’s tests. *T*-tests were performed between cabinets with the same treatment combination, and no effects of the growth cabinet were observed. In all analyses, test results were considered to be significant at *P <* 0.05. A Bonferroni post hoc test was used to compare group means, where ANOVA determined significant effects. A repeated measures ANOVA was performed through the aov function in the stats R package to evaluate the effects of [CO_2_], temperature and genotype over time on chlorophyll content. For leaf length, a four-parameter generalized logistic function was used to describe the growth increase over time by using the drm function from R package drc (Ritz et al. 2015) according to the following equation: 


\begin{equation*} W= \frac{a+d}{1+\exp \left(-b\left(T-c\right)\right)},\end{equation*}

where *W* is leaf length, *T* is time in days, *a* is the upper asymptote of leaf growth, *d* is the lower asymptote of leaf growth, *c* is the time (*T*) value with a response half-way between *a* and *d*, while *b* is the correspondent slope around the inflexion point. Regressions were performed across the treatments, and the maximum leaf length and time to reach 95% of the maximum leaf length were calculated from the equation. Subsequently, effects of genotype, temperature and [CO_2_] on these parameters were compared using ANOVA. For flushing interval, number of leaves per flush, SD, leaf nitrogen concentration and leaf carbon:nitrogen ratio (C:N), a three-way ANOVA was used to test the main effects of [CO_2_], temperature and genotype and their interaction using aov function from the stats R package. To test the treatment effects on the gas exchange parameters (*P*_n_, *E*, *g*_s_ and *i*WUE) and *F*_v_*/F*_m_ and PI, a linear mixed-effect model was employed using the lmer function from the nlme R package (Pinheiro et al. 2023) with [CO_2_], temperature and genotypes as fixed factors and day of measurement as a random factor.

## Results

### Photosynthesis and gas exchange parameters

**Figure 2:**
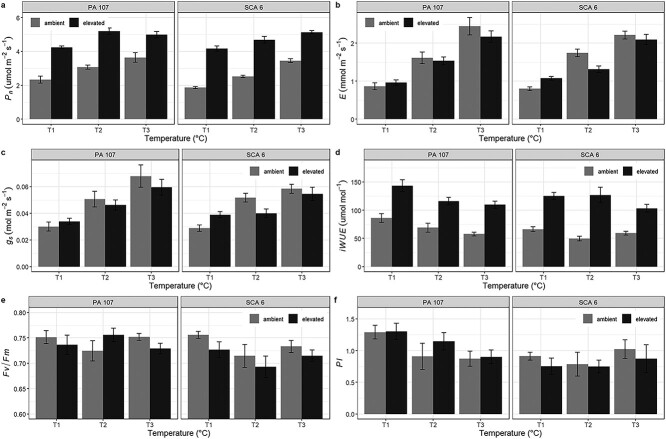
Light-saturated net photosynthesis rate (*P*_n_) (a), transpiration rate (*E*) (b), stomatal conductance (*g*_s_) (c), *i*WUE (d), maximum quantum efficiency (*F*_v_*/F*_m_) (e) and PI (f) measured on two juvenile cacao genotypes grown under two [CO_2_] and three temperatures. Error bars show the standard error of the mean (*n* = 6). [CO_2_] treatments are ambient (grey bar) and elevated (black bar). Temperature treatments are 31/22 °C (T1), 33.5/24.5 °C (T2) and 36/27 °C (T3).

Overall, light-saturated net photosynthesis rate (*P*_n_) was slightly higher in PA 107 (3.91 (±0.14) μmol m^−2^ s^−1^) compared with SCA 6 (3.64 (±0.11) μmol m^−2^ s^−1^) (*P* < 0.05; Figure 2a). Elevated [CO_2_] had a positive effect on *P*_n_ in both genotypes (*P* < 0.001); the increase was 69% greater in plants grown at elevated compared with ambient [CO_2_]. A significant increase of 23 and 37% in *P*_n_ was observed with an increase in temperature from 31/22 to 33.5/24.5 and 36/27 °C, respectively (*P* < 0.001). There was no significant interaction between the treatments for *P*_n_.

Leaf transpiration (*E*) increased by 67% from 31/22 to 33.5/24.5 °C and 141% from 31/22 to 36/27 °C (*P* < 0.001), while *g*_s_ increased by 43 and 82% across the same temperature range (*P* < 0.001) (Figure 2b and c). At the two highest temperatures, *E* and *g*_s_ declined under elevated [CO_2_]; the interaction between temperature and [CO_2_] was significant for *E* (*P* < 0.05). No differences between genotypes or other interactions for *E* and *g*_s_ were observed. Overall, *i*WUE was higher in PA 107 (96.98 (±3.31) μmol mol^−1^) compared with SCA 6 (88.44 (±3.19) μmol mol^−1^) (*P* < 0.05; Figure 2d). There was a positive effect of elevated [CO_2_] on *i*WUE in both genotypes (86% higher in plants grown at elevated compared with ambient [CO_2_] (*P* < 0.001). Overall, an increase in temperature from 31/22 to 36/27 °C resulted in a decline in *i*WUE by 22% (*P* < 0.001). There was no significant interaction among treatments for *i*WUE (Figure 2d).

A slightly higher *F*_v_*/F*_m_ ratio was observed for PA 107 compared with SCA 6 (0.74 (±0.01) and 0.72 (±0.01), respectively) (*P* < 0.05) (Figure 2e). Similarly, PI was greater in PA 107 (1.07 (±0.07)) compared with SCA 6 (0.85 (±0.07)) (*P* < 0.01) (Figure 2f). There were no significant effects of [CO_2_] or temperature on *F*_v_*/F*_m_ and PI.

### Plant growth and leaf traits

**Figure 3:**
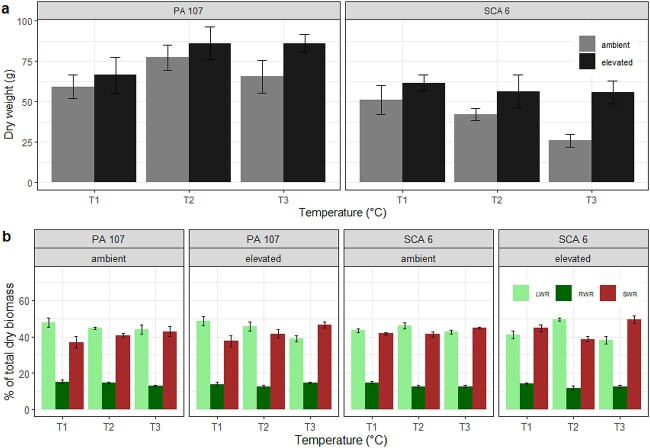
Final dry weight (a) and dry weight allocation (b) measured on two juvenile cacao genotypes grown under two [CO_2_] and three temperatures. Error bars show the standard error of the mean (*n* = 6). In (a), [CO_2_] treatments are ambient (grey bar) and elevated (black bar). Temperature treatments are 31/22 °C (T1), 33.5/24.5 °C (T2) and 36/27 °C (T3). In (b), bars represent leaf weight ratio (LWR—light green), stem weight ratio (SWR—dark green) and root weight ratio (RWR—brown).

Elevated [CO_2_] had a positive effect on final plant dry weight (*P* < 0.01). Plants grown at elevated [CO_2_] accumulated, on average, 29% more dry weight than those grown under ambient [CO_2_] (Figure 3a). The effect of temperature on plant dry weight varied between genotypes (*P* < 0.05 for temperature^*^genotype interaction). In PA 107, final plant dry weight was 30% greater at 33.5/24.5 °C compared with the control; with a further increase in temperature, the changes in plant dry weight were not significant. By contrast, in SCA 6, the final plant dry weight was lower at 33.5/24.5 °C (12%) and 36/27 °C (28%) compared with the control (31/22 °C), particularly at ambient [CO_2_]. There was no significant interaction between [CO_2_] and temperature for either genotype (Figure 3a).

Overall, the below-ground dry weight allocation declined significantly with increased temperature (*P* < 0.05) (Figure 3b). Root weight ratio (root dry weight/total plant dry weight; Hunt et al. 2002) declined by 11% as temperature increased from 31/22 to 33.5/24.5 °C; no further changes in root weight ratio were observed at 36/27 °C. Increased temperature resulted in a 13% reduction in root:shoot ratio (data not shown); no clear effects of [CO_2_] or genotype were noted on root:shoot ratio. The effect of temperature on leaf and stem weight ratio (leaf dry weight/total plant dry weight, stem dry weight/total plant dry weight) varied between genotypes (*P* < 0.05 and *P* < 0.01, respectively) (Figure 3b). In PA 107, there was a decline in leaf weight ratio and an increase in stem weight ratio with increasing temperature. In SCA 6, there were no clear trends in stem or leaf weight ratio with increasing temperature. There were no significant effects of [CO_2_] on dry weight allocation in either genotype (Figure 3b).

**Figure 4:**
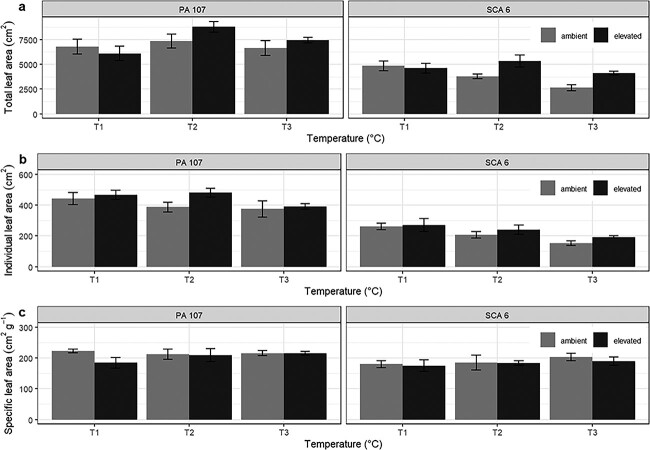
Total leaf area (a), individual leaf area (b) and SLA (c) measured on two juvenile cacao genotypes grown under two [CO_2_] and three temperatures. Error bars show the standard error of the mean (*n* = 6). [CO_2_] treatments are ambient (grey bar) and elevated (black bar). Temperature treatments are 31/22 °C (T1), 33.5/24.47 °C (T2) and 36/27 °C (T3).

Leaf area showed a similar response to the treatments as total dry weight. The genotype PA 107 had a significantly higher final leaf area (*P* < 0.001) (Figure 4a) than SCA 6 (7191.5 (±283.3) cm^2^ and 4224.9 (±215.8) cm^2^, respectively; Figure 4a). The effect of temperature on final leaf area differed between genotypes (*P* < 0.05 for temperature^*^genotype interaction; Figure 4a). In SCA 6, the leaf area declined with increasing temperature at ambient [CO_2_]. However, in PA 107, the leaf area increased by 25% at 33.5/24.5 °C compared with the control with no further significant change at 36/27 °C. There was also a significant interaction between temperature and [CO_2_] on the final leaf area (*P* < 0.05; Figure 4a); greater leaf area at elevated [CO_2_] was evident at the two higher temperatures, whereas at the control temperature, no difference between [CO_2_] treatments was observed.

Mean individual leaf area was significantly higher in PA 107 compared with SCA 6 (423.6 (±14.9) and 220.5 (±11.9) cm^2^, respectively; *P* < 0.001; Figure 4b). Increasing temperature from 31/22 to 36/27 °C resulted in a 22% overall decrease in individual leaf area (*P* < 0.01). Conversely, elevated [CO_2_] resulted in a 12% increase in individual leaf area (*P* < 0.05) (Figure 4b). There were no interactions between treatments in relation to the individual leaf area. The SLA was not affected by [CO_2_] and temperature treatments but was higher in PA 107 than SCA 6 (210.1 (±5.7) and 185.9 (±6.1) cm^2^ g^−1^, respectively) (*P* < 0.01) (Figure 4c).

**Figure 5:**
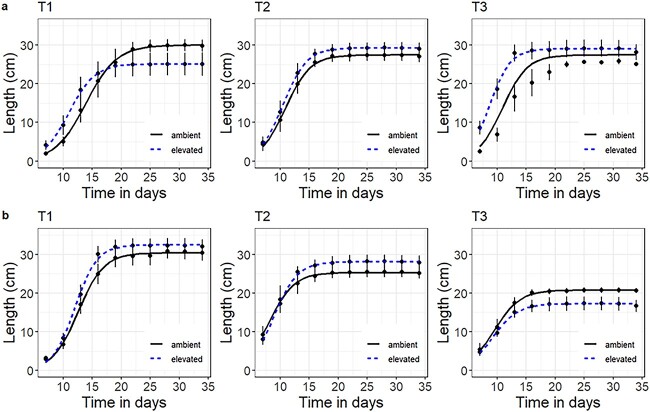
Increase in leaf length for PA 107 (a) and SCA 6 (b) grown under two [CO_2_] and three temperature regimes. Curves based on four-parameter generalized logistic equations were applied to each treatment combination (*n* = 6). Temperature treatments are T1—31/22 °C, T2—33.5/24.5 °C and T3—36/27 °C. [CO_2_] treatments are ambient (black solid lines) and elevated (blue dashed lines).

The increase in leaf length for each treatment combination for both genotypes is shown in Figure 5, and the final leaf length reached (fitted logistic regression parameter ‘*d*’) is presented in Table 2. A significant interaction between genotype and temperature was observed on the final leaf length (*P* < 0.001). In SCA 6, the final leaf length decreased with increasing temperature, while for PA 107, the final leaf length was unaffected by temperature. The effect of [CO_2_] on leaf length was inconsistent between genotypes and temperature (Figure 5). For example, at 36/27 °C, the leaf length was higher at elevated [CO_2_] for PA 107, whereas for SCA 6, it was higher at ambient [CO_2_]. For both genotypes, the time to reach 95% of full leaf size was reduced as temperature increased (*P* < 0.001; Table 2). A significant interaction between [CO_2_] and genotypes was observed (*P* < 0.05) such that, for PA 107, the time to reach 95% of the maximum leaf length decreased significantly from 16.9 (±0.9) days at ambient [CO_2_] to 13.6 (±0.7) days at elevated [CO_2_], whereas for SCA 6, there were no significant differences between CO_2_ treatments.

**Table 2:** The effect of [CO_2_] and temperature on flushing interval (FI), number of leaves per flush, SD, leaf N concentration, leaf carbon:nitrogen ratio (C:N), maximum leaf length (MaxLL) and time to reach 95% of the maximum leaf length (Time95L) of two juvenile cacao genotypes.

Genotype	Treatment		FI (days)	Leaves per flush	SD (stomata mm^−2^)	N (%)	C:N	MaxLL (cm)	Time95L (days)
	Temp (°C)	[CO_2_]							
PA 107	31/22	Ambient	31 (±1.1)	14 (±0.8)	995 (±12.3)	2.7 (±0.06)	17.1 (±0.35)	30.0 (±1.5)	19 (±1.5)
		Elevated	29 (±1.2)	13 (±1.1)	888 (±24.8)	2.6 (±0.07)	17.9 (±0.37)	26.7 (±1.7)	16 (±1.6)
	33.5/24.5	Ambient	26 (±0.8)	19 (±1.7)	1143 (±49.7)	2.5 (±0.10)	18.6 (±0.71)	27.3 (±1.4)	16 (±0.5)
		Elevated	28 (±0.8)	18 (±2.3)	1015 (±36.4)	2.4 (±0.10)	19.6 (±0.84)	29.1 (±1.4)	14 (±1.0)
	36/27	Ambient	24 (±0.9)	18 (±1.4)	984 (±47.1)	2.5 (±0.08)	18.3 (±0.66)	25.5 (±0.4)	16 (±2.0)
		Elevated	24 (±0.6)	19 (±1.5)	938 (±36.2)	2.7 (±0.07)	16.9 (±0.42)	28.9 (±2.1)	12 (±0.4)
SCA 6	31/22	Ambient	33 (±0.8)	19 (±1.6)	1274 (±42.2)	2.7 (±0.01)	17.1 (±0.11)	30.8 (±2.1)	17 (±0.7)
		Elevated	35 (±1.7)	18 (±1.1)	1107 (±48.1)	2.2 (±0.06)	21.8 (±0.62)	32.2 (±1.8)	17 (±0.4)
	33.5/24.5	Ambient	28 (±0.8)	19 (±0.9)	1486 (±39.8)	2.1 (±0.07)	22.7 (±0.7)	25.4 (±1.3)	13 (±1.4)
		Elevated	27 (±0.8)	23 (±1.5)	1431 (±17.3)	2.0 (±0.03)	23.4 (±0.39)	28.1 (±1.7)	13 (±1.3)
	36/27	Ambient	27 (±0.7)	18 (±1.1)	1347 (±32.6)	2.4 (±0.04)	19.7 (±0.28)	20.6 (±0.6)	14 (±1.0)
		Elevated	24 (±1.2)	21 (±0.4)	1213 (±24.2)	2.2 (±0.09)	22.1 (±0.97)	17.1 (±1.7)	14 (±0.7)
Statistics									
Temp			^*^^*^^*^	^*^^*^^*^	^*^^*^^*^	^*^^*^^*^	^*^^*^^*^	^*^^*^^*^	^*^^*^^*^
CO_2_			ns	ns	^*^^*^^*^	^*^^*^^*^	^*^^*^^*^	ns	^*^^*^
Temp^*^CO_2_			ns	ns	ns	^*^^*^	^*^^*^	ns	ns
Gen			^*^^*^	^*^^*^	^*^^*^	^*^^*^^*^	^*^^*^^*^	^*^	ns
Gen^*^Temp			ns	ns	ns	ns	ns	^*^^*^^*^	ns
Gen^*^CO_2_			ns	ns	ns	^*^^*^	^*^^*^	ns	^*^
Gen^*^Temp^*^CO_2_			ns	ns	ns	^*^	^*^	ns	ns

Values are means (± standard errors, *n* = 6). In the statistics, Temp: temperature; CO_2_: [CO_2_]; Gen: genotype. Asterisks (^*^) indicate significant differences (^*^P < 0.05, ^*^^*^P < 0.01, ^*^^*^^*^P < 0.001); ns indicates non-significant differences (*P* > 0.05).

Flushing interval and the number of leaves per flush were not affected by elevated [CO_2_] (Table 2). Flushing interval decreased from 32 (±1.2) days at 31/22 °C to 27 (±0.8) and 25 (±0.8) days at 33.5/24.5 and 36/27 °C, respectively (*P* < 0.001). Flushing interval was, on average, 2 days longer in SCA 6 in comparison with PA 107 (*P* < 0.001). There were no significant interactions between treatments in relation to flushing interval. Overall, PA 107 produced fewer leaves per flush (17 (±1)) than SCA 6 (20 (±1)) (*P* < 0.001; Table 2). Leaf number per flush also increased from 16 (±1) at 31/22 °C to 20 (±1) leaves at 33.5/24.5 °C (±1) and 19 (±1) leaves at 36/27 °C (*P* < 0.01). There were no significant interactions between the treatments on leaves per flush. The SD was significantly lower in PA 107 (994 (±20) stomata mm^−2^ compared with SCA 6 (1310 (±26) stomata mm^−2^) (*P* < 0.001). There was also an overall reduction in SD from 1205 (±34) stomata mm^−2^ in plants grown at ambient [CO_2_] to 1099 (±34) stomata mm^−2^ in plants grown at elevated [CO_2_] (*P* < 0.001). The SD did not differ between 31/22 °C (1066 (±35.1) stomata mm^−2^) and 36/27 °C (1121 (±37.5) stomata mm^−2^) but was significantly higher (*P* < 0.01) in both genotypes (1269 (±45) stomata.mm^−2^) at 33.5/24.5 °C. There were no significant interactions between treatments on SD (Table 2).

A significant interaction was observed between [CO_2_], temperature and genotype for leaf nitrogen concentration (*P* < 0.05) (Table 2). For PA 107, leaf nitrogen concentration was significantly higher at 31/22 °C (2.67 (±0.04) %) compared with 33.5/24.5 °C (2.44 (±0.07) %), whereas no effect of elevated [CO_2_] was observed. By contrast, for SCA 6 differences in leaf N concentration across the temperature treatments varied according to [CO_2_]. At ambient [CO_2_], the highest nitrogen concentration was observed at 31/22 °C (2.74 (±0.01) % compared with 2.08 (±0.07) % and 2.42 (±0.04) % at 33.5/24.5 and 36/27 °C, respectively). At elevated [CO_2_], no differences in the leaf N concentration across the temperatures were observed. A significant interaction between [CO_2_], temperature and genotype was also observed for the leaf C:N ratio (*P* < 0.05) (Table 2). In PA 107, C:N was higher at 33.5/24.5 °C (19.1 (±0.5)) compared with 31/22 and 36/27 °C (17.5 (±0.3) and 17.6 (±0.4), respectively), while no significant effect of elevated [CO_2_] was observed. However, in SCA 6, C:N in plants grown at elevated [CO_2_] increased by 27% (at 31/22 °C) and 11% (at 36/27 °C) compared with those grown at ambient [CO_2_], whereas no effects of elevated [CO_2_] was observed at 33.5/24.5 °C.

Leaf chlorophyll content increased over the experimental period (*P* < 0.001) on average from 13.1 (±0.1) μg cm^−2^ at 10 days after emergence to 36.2 (±1.1) μg cm^−2^ at 46 days after emergence (see Figure S2 available as Supplementary data at *Tree Physiology* Online). Overall, leaf chlorophyll content was significantly higher for PA 107 compared with SCA 6 (*P* < 0.001) (27.5 (± 0.5) and 23.9 (±0.3) μg cm^−2^, respectively). This difference was statistically significant from Day 31 (*P* < 0.05). There were no differences between temperature regimes, [CO_2_] or their interaction on leaf chlorophyll content.

## Discussion

### Effect of temperature and elevated [CO_2_] on photosynthetic traits

The increase in photosynthetic rate and stomatal conductance across the range of temperatures studied up to 36/27 °C, combined with the maintenance of chlorophyll fluorescence parameters (*F*_v_*/F*_m_ and PI), suggests that supra-optimal temperatures for cacao were not experienced in this study. This was contrary to our hypothesis based on earlier studies that have reported optimum temperatures for net photosynthesis in cacao of 33 °C (Balasimha et al. 1991) above which there is a decline. Such a decline is thought to occur, in part, due to increases in respiration and/or stomatal closure as a protective mechanism to reduce water loss in response to the increased evaporative demand experienced with increases in temperature. Previous studies in cacao have shown that photosynthesis declines as VPD increases with temperature (Raja Harun and Hardwick 1988, Hernandez et al. 1989, Baligar et al. 2008), which is coupled with decreased stomatal conductance (Sena Gomes and Kozlowski 1987, Raja Harun and Hardwick 1988, Hernandez et al. 1989, Baligar et al. 2008). In the present study, VPD was maintained constant across the temperature treatments (0.9 kPa) in order to remove the confounding effect of VPD and to explore the direct effects of temperature. These results suggest that the previously reported optimum temperature range for photosynthesis in cacao is likely to have been misinterpreted due to the confounding effect of VPD. More studies are required to understand the impact of VPD on photosynthetic functioning in cacao in combination with different temperatures. Due to the short-term nature of the current study (88 days), thermal acclimation to the highest temperature was not considered; however, this should be included in future longer-term studies.

Irrespective of temperature, photosynthesis increased significantly in plants grown at elevated [CO_2_] compared with those grown under ambient conditions; the average increase observed here of 68% (Figure 2a) is somewhat higher than the range of 10–56% reported in other studies (Lahive et al. 2018, Hebbar et al. 2020, Baligar et al. 2021*a*). However, *g*_s_ and *E* declined in plants grown under elevated [CO_2_] at the higher temperature regimes. This is largely consistent with Drake et al. (1997) (albeit most notable at the higher temperatures) whose meta-analysis indicated this feature in plants grown under short-term [CO_2_] enrichment. The observed increase in *i*WUE at elevated [CO_2_] was driven by the reduction in stomatal conductance. Increases in WUE at elevated [CO_2_] have previously been observed in cacao seedlings (Lahive et al. 2018, Hebbar et al. 2020, Baligar et al. 2021*a*). Here, we have shown that in spite of the small decreases in *i*WUE with increased temperatures, this was counter-balanced by the effect of elevated [CO_2_]. Therefore, elevated [CO_2_] could potentially improve the water status of cacao plants under warmer conditions.

Leaf traits, such as SD and nitrogen content, can potentially influence photosynthetic performance. The SD was highest at the intermediate temperature of 33.5/24.5 °C (Table 2). Increases in SD have been considered to be an adaptive mechanism to the increased evaporative demand in warm environments (Jumrani et al. 2017), while reductions in SD have resulted from morphological adjustments in order to prevent water loss at the highest temperatures (Caine et al. 2019). Such a reduction in SD might also lead to decreases in stomatal conductance and photosynthesis rates (Xu and Zhou 2008). However, in this study, SD was not correlated with gas exchange parameters. The constant VPD maintained across the temperature treatments in this study may account for this lack of correlation. The observation of a 9% decrease in SD in leaves of plants grown at elevated [CO_2_] is consistent with a survey conducted by Woodward and Kelly (1995) who showed that, in many species, there was a reduction in SD under elevated [CO_2_]. However, SD responses to elevated [CO_2_] in cacao have not shown a conclusive trend. Increases in SD of ~9% were seen in leaves of young Amelonado cacao plants grown at elevated [CO_2_] under glasshouse conditions, while there was no overall change observed in six mature cacao clones grown under similar conditions (Lahive et al. 2018, 2021).

The effect of elevated [CO_2_] and temperature on leaf C:N differed between the two genotypes (Table 2), these changes being driven by leaf nitrogen content. Similar genotypic differences in the leaf nitrogen content have also been noted among a set of eight cacao clones grown under greenhouses conditions (Daymond et al. 2011). Here, the changes in the leaf nitrogen content may have resulted in the variation in chlorophyll content observed between genotypes (see Figure S2 available as Supplementary data at *Tree Physiology* Online). In this study, irrespective of genotype, the leaf nitrogen content decreased in plants grown at elevated [CO_2_]. Previous studies have shown similar reductions in the leaf nitrogen content in response to elevated [CO_2_] in cacao (Lahive et al. 2018) and other species (Coleman et al. 1993, Feng et al. 2015, Ainsworth and Long 2021). This reduction has been explained as a dilution effect of accumulated non-structural carbohydrates from the increased photosynthesis (Ainsworth and Long 2005, Sun et al. 2012). Uddling et al. (2018) provide further explanation for the reduced leaf nitrogen concentration at elevated CO_2_, including decreased Rubisco demand, decreased transpiration-driven mass flow of N towards roots and inhibited shoot nitrate assimilation. However, the fact that photosynthesis increased at elevated [CO_2_] despite the decline in leaf nitrogen may indicate enhanced nitrogen-use efficiency under elevated [CO_2_]. Increased nitrogen-use efficiency is associated with greater Rubsico efficiency, with less nitrogen investment required to achieve similar or higher photosynthesis rates (Leakey et al. 2009). Thus, this can be important in particular growing regions where cacao is cultivated under low-fertility soils.

### Effect of elevated [CO_2_] and temperature on growth and biomass

Independent effects of temperature and [CO_2_] on the dry weight and leaf area have been reported in young cacao plants, with significant reductions when temperature increases above an optimal level for growth (Sale 1968, Sena Gomes and Kozlowski 1987, Hebbar et al. 2020), and there are significant enhancements with increasing [CO_2_] (Baligar et al. 2005, 2021*a*, 2021*b*, Lahive et al. 2018, Hebbar et al. 2020). Here, evidence was found for different sensitivities to increasing temperature between the two genotypes; at the highest temperature and at ambient [CO_2_], the dry weight and leaf area of SCA 6 decreased relative to the control temperature, whereas little change was evident in PA 107. However, while dry weight and leaf area increased at higher temperatures and at elevated [CO_2_] in PA 107, in SCA 6, the negative effect of the highest temperature observed at ambient [CO_2_] was compensated by exposure to elevated [CO_2_]. A compensatory effect of [CO_2_] to high temperature was reported by Hebbar et al. (2020), working on a single genotype. Here, the results suggest that [CO_2_] elevation within the range predicted in the latter part of this century may ameliorate the negative impact of higher temperatures in some genotypes and stimulate growth in others. While no clear effects of elevated [CO_2_] were observed in dry weight allocation patterns (Figure 3b), the root:shoot ratio declined at the highest temperature. Sena Gomes and Kozlowski (1987) also previously reported a decrease in root:shoot ratio when cacao seedlings were grown at temperatures above 22.2 °C. The fact that we have also observed such a shift in dry weight allocation with increased temperatures under elevated [CO_2_] implies that young cacao plants may be less adapted to future climate change conditions if water and nutrients are limiting.

Overall, the increase in dry weight and leaf area under elevated [CO_2_] was not as great as that of photosynthesis. Although not measured here, leaf respiration has been shown to be higher in cacao at elevated [CO_2_] (Lahive et al. 2021) and so may explain this disparity. Similarly, higher respiration rates often observed at higher temperatures may also have been a factor in the lack of correlation between the generally positive effects of increased temperature on photosynthesis and its negative or neutral impact on growth (Dusenge et al. 2019).

The two genotypes also exhibited different morphological responses to increased temperature; much larger reductions in the final leaf length were observed for SCA 6 compared with PA 107 (Figure 4). It has been suggested that plants at elevated temperatures tend to produce smaller leaves in order to offset the water loss due to the higher transpiration (Qaderi et al. 2006) or as a thermoregulatory adaptive trait (Tserej and Feeley 2021). Despite higher temperature leading to the production of smaller leaves, leaf growth rate was higher (especially in PA 107) with leaves reaching their final length more quickly under warmer conditions (Table 2). The faster rate of leaf production at higher temperatures was coupled with a reduction in flushing interval. Previous observations in cacao have demonstrated a reduction in flushing interval, with increases in temperature both in controlled environment growth chambers (Sale 1968) and under field conditions (Sena Gomes et al. 1987, De Almeida and Valle 2007). Similar observations have been made for some tropical fruits (Menzel and Simpson 1988, Utsunomiya 1992). Here, we have shown that such a reduction is maintained at elevated [CO_2_].

## Conclusions

We provide evidence that the interaction between elevated [CO_2_] and increasing temperatures on the growth and physiological responses of two cacao genotypes indicates the importance of considering multiple factors when assessing the impacts of climate change on crop performance. We have shown that, in the absence of other stresses, notably VPD, photosynthetic rate responds positively to higher temperatures than previously reported in cacao. However, the impact of temperature and [CO_2_] on growth varied between the two genotypes studied here, highlighting the influence of genotype in response to climate change and the importance of evaluating a range of germplasm under future climate scenarios. The results imply that, under non-limiting water and nutrient conditions, elevated [CO_2_] increases biomass production in juvenile cacao plants under warmer conditions for high-temperature-tolerant cacao genotypes and compensates for the negative effects of a temperature increase (5 °C above current West African conditions) for more temperature-sensitive genotypes.

## Supplementary Material

TP-2023_109_Supplementary_tpad116

## Data Availability

The data that support the findings of this study are available from the corresponding author upon reasonable request.

## References

[ref1] Ainsworth EA, Long SP (2005) What have we learned from 15 years of free-air CO_2_ enrichment (FACE)? A meta-analytic review of the responses of photosynthesis, canopy properties and plant production to rising CO_2_. New Phytol 165:351–372. 15720649 10.1111/j.1469-8137.2004.01224.x

[ref2] Ainsworth EA, Long SP (2021) 30 years of free-air carbon dioxide enrichment (FACE): What have we learned about future crop productivity and its potential for adaptation? Glob Chang Biol 27:27–49. 33135850 10.1111/gcb.15375

[ref3] Balasimha D, Daniel EV, Bhat PG (1991) Influence of environmental factors on photosynthesis in cocoa trees. Agric For Meteorol 55:15–21.

[ref4] Baligar VC, Bunce JA, Bailey BA, Machado RCR, Pomella AWV (2005) Carbon dioxide and photosynthetic photon flux density effects on growth and mineral uptake of cacao. J Food Agric Environ 3:142–147.

[ref5] Baligar VC, Bunce JA, Machado RCR, Elson MK (2008) Photosynthetic photon flux density, carbon dioxide concentration, and vapor pressure deficit effects on photosynthesis in cacao seedlings. Photosynthetica 46:216–221.

[ref6] Baligar VC, Elson MK, Almeida A-AF, de Araujo QR, Ahnert D, He Z (2021 *a*) The impact of carbon dioxide concentrations and low to adequate photosynthetic photon flux density on growth, physiology and nutrient use efficiency of juvenile cacao genotypes. Agronomy 11:397. 10.3390/agronomy11020397.

[ref7] Baligar VC, Elson MK, Almeida A-AF, de Araujo QR, Ahnert D, He Z (2021 *b*) Carbon dioxide concentrations and light levels on growth and mineral nutrition of juvenile cacao genotypes. Am J Plant Sci 12:818–839.

[ref8] Caine RS, Yin X, Sloan J et al. (2019) Rice with reduced stomatal density conserves water and has improved drought tolerance under future climate conditions. New Phytol 221:371–384. 30043395 10.1111/nph.15344 PMC6492113

[ref9] Coleman JS, McConnaughay KDM, Bazzaz FA (1993) Elevated CO_2_ and plant nitrogen-use: Is reduced tissue nitrogen concentration size-dependent? Oecologia 93:195–200. 28313607 10.1007/BF00317671

[ref10] Conroy JP, Milham PJ, Mazur M, Barlow EWR (1990) Growth, dry weight partitioning and wood properties of *Pinus radiata* D. Don after 2 years of CO_2_ enrichment. Plant Cell Environ 13:329–337.

[ref11] DaMatta FM, Avila RT, Cardoso AA, Martins SCV, Ramalho JC (2018) Physiological and agronomic performance of the coffee crop in the context of climate change and global warming: a review. J Agric Food Chem 66:5264–5274. 29517900 10.1021/acs.jafc.7b04537

[ref12] Daymond AJ, Hadley P (2004) The effects of temperature and light integral on early vegetative growth and chlorophyll fluorescence of four contrasting genotypes of cacao (*Theobroma cacao*). Ann Appl Biol 145:257–262.

[ref13] Daymond AJ, Tricker PJ, Hadley P (2011) Genotypic variation in photosynthesis in cacao is correlated with stomatal conductance and leaf nitrogen. Biol Plant 55:99–104.

[ref14] De Almeida A-AF, Valle RR (2007) Ecophysiology of the cacao tree. Braz J Plant Physiol 19:425–448.

[ref15] Drake BG, Gonzàlez-Meler MA, Long SP (1997) More efficient plants: a consequence of rising atmospheric CO_2_? Annu Rev Plant Physiol Plant Mol Biol 48:609–639. 15012276 10.1146/annurev.arplant.48.1.609

[ref16] Dusenge ME, Duarte AG, Way DA (2019) Plant carbon metabolism and climate change: elevated CO_2_ and temperature impacts on photosynthesis, photorespiration and respiration. New Phytol 221:32–49. 29983005 10.1111/nph.15283

[ref17] End MJ (1990) A study of the effects of the photo-thermal environment on fruit and seed growth and development in *Theobroma cacao* L. PhD thesis. University of Reading, UK.

[ref18] Feng Z, Rütting T, Pleijel H et al. (2015) Constraints to nitrogen acquisition of terrestrial plants under elevated CO_2_. Glob Chang Biol 21:3152–3168. 25846203 10.1111/gcb.12938

[ref19] Guillou C, Fillodeau A, Brulard E, Breton D, De Faria MS, Verdier D, Simon M, Ducos J-P (2018) Indirect somatic embryogenesis of *Theobroma cacao* L. in liquid medium and improvement of embryo-to-plantlet conversion rate. In Vitro Cell Dev Biol Plant 54:377–391. 30147286 10.1007/s11627-018-9909-y PMC6096749

[ref20] Hadley P, End MJ, Taylor SJ, Pettipher GL (1994) Environmental regulation of vegetative and reproductive growth in cocoa grown in controlled environment glasshouse conditions. In: Tay EB, Lee MT, Yap TN, Zulkarnain BI, Thong FT, Bong SL, Tee SK (eds) Proceedings of the International Cocoa Conference: Challenges in the 90s. Kuala Lumpur, Malaysia, Malaysian Cocoa Board, pp 319–331.

[ref21] Hatfield JL, Prueger JH (2015) Temperature extremes: effect on plant growth and development. Weather Clim Extrem 10:4–10.

[ref22] Hebbar KB, Apshara E, Chandran KP, Prasad Vara PV (2020) Effect of elevated CO_2_, high temperature, and water deficit on growth, photosynthesis, and whole plant water use efficiency of cocoa (*Theobroma cacao* L.). Int J Biometeorol 64:47–57. 31468175 10.1007/s00484-019-01792-0

[ref23] Hernandez A, Cock J, El-Sharkawy M (1989) The responses of stomatal conductance to air humidity in shade-grown coffee, tea and cacao plants as compared with sunflower. Rev Bras Fisiol Veg 1:155–161.

[ref24] Hunt R, Causton DR, Shipley B, Askew AP (2002) A modern tool for classical plant growth analysis. Ann Bot 90:485–488. 12324272 10.1093/aob/mcf214 PMC4240380

[ref25] ICCO (2023) Quarterly bulletin of cocoa statistics, vol. XLVII – No. 4 – cocoa year 2020/2021. www.icco.org.

[ref26] IPCC (2021) Technical summary. In: Masson-Delmotte V et al. (eds) Climate change 2021: the physical science basis. Contribution of working group I to the sixth assessment report of the Intergovernmental Panel on Climate Change. Cambridge University Press, Cambridge and New York, pp 33–144.

[ref27] Jumrani K, Bhatia VS, Pandey GP (2017) Impact of elevated temperatures on specific leaf weight, stomatal density, photosynthesis and chlorophyll fluorescence in soybean. Photosynth Res 131:333–350. 28025729 10.1007/s11120-016-0326-y

[ref29] Krause GH, Winter KA, Krause B, Virgo A (2015) Light-stimulated heat tolerance in leaves of two neotropical tree species, *Ficus insipida* and *Calophyllum longifolium*. Funct Plant Biol 42:42–51. 10.1071/FP14095 32480652

[ref30] Kumari M, Verma S, Bhardwaj S (2019) Effect of elevated CO_2_ and temperature on growth and yield contributing parameters of pea (*Pisum sativum* L.) crop. J Agrometeorol 21:7–11.

[ref31] Lahive F (2015) An examination of the impacts of climate change variables on growth and photosynthesis in *Theobroma cacao* L.. PhD thesis. University of Reading, UK.

[ref32] Lahive F, Hadley P, Daymond AJ (2018) The impact of elevated CO_2_ and water deficit stress on growth and photosynthesis of juvenile cacao (*Theobroma cacao* L.). Photosynthetica 56:911–920.

[ref33] Lahive F, Hadley P, Daymond AJ (2019) The physiological responses of cacao to the environment and the implications for climate change resilience. A review. Agron Sustain Dev 39:1–22.30881486

[ref34] Lahive F, Handley LR, Hadley P, Daymond AJ (2021) Climate change impacts on cacao: genotypic variation in responses of mature cacao to elevated CO_2_ and water deficit. Agronomy 11:818. 10.3390/agronomy11050818.

[ref35] Leakey ADB, Ainsworth EA, Bernacchi CJ, Rogers A, Long SP, Ort DR (2009) Elevated CO_2_ effects on plant carbon, nitrogen, and water relations: six important lessons from FACE. J Exp Bot 60:2859–2876. 19401412 10.1093/jxb/erp096

[ref36] Lee JS (2011) Combined effect of elevated CO_2_ and temperature on the growth and phenology of two annual C_3_ and C_4_ weedy species. Agric Ecosyst Environ 140:484–491.

[ref37] Lima LJR, Almeida MH, Nout MJR, Zwietering MH (2011) *Theobroma cacao* L., “the food of the gods”: quality determinants of commercial cocoa beans, with particular reference to the impact of fermentation. Crit Rev Food Sci Nutr 51:731–761. 21838556 10.1080/10408391003799913

[ref38] Mensah EO, Asare R, Vaast P, Amoatey CA, Markussen B, Owusu K, Asitoakor BK, Ræbild A (2022) Limited effects of shade on physiological performances of cocoa (*Theobroma cacao* L.) under elevated temperature. Environ Exp Bot 201:104983. 10.1016/j.envexpbot.2022.104983.

[ref39] Menzel CM, Simpson DR (1988) Effect of temperature on growth and flowering of litchi (*Litchi chinensis* Sonn.) cultivars. J Hortic Sci 63:349–360.

[ref40] Motamayor JC, Lachenaud P, da Silva e Mota JW, Loor R, Kuhn DN, Brown JS, Schnell RJ (2008) Geographic and genetic population differentiation of the Amazonian chocolate tree (*Theobroma cacao* L.). PloS One 3:1–8. 10.1371/journal.pone.0003311 PMC2551746 18827930

[ref41] Pinheiro J, Bates D, DebRoy S, Sarkar D (2023) nlme: linear and nonlinear mixed effects models. https://cran.r-project.org/package=nlme (29 June 2023, date last accessed).

[ref42] Qaderi MM, Kurepin LV, Reid DM (2006) Growth and physiological responses of canola (*Brassica napus*) to three components of global climate change: temperature, carbon dioxide and drought. Physiol Plant 128:710–721.

[ref43] R Core Team (2021) R: a language and environment for statistical computing. R Foundation for Statistical Computing, Vienna, Austria.

[ref44] Raja Harun RM, Hardwick K (1988) The effect of different temperatures and water-vapor pressure deficits on photosynthesis and transpiration of cocoa leaves. In: Proceedings of the 10th International Cocoa Research Conference. Cocoa Producers’ Alliance, Santo Domingo, Dominican Republic, pp 211–214.

[ref45] Ríos-Bolívar FM, Garruña R, Rivera-Hernández B, Herrera A, Tezara W (2022) Effect of high concentrations of CO_2_ and high temperatures on the physiology of Mexican cocoa. Plant Stress 6:100114. 10.1016/j.stress.2022.100114.

[ref46] Ritz C, Baty F, Streibig JC, Gerhard D (2015) Dose-response analysis using R. PloS One 10:1–13. 10.1371/journal.pone.0146021 PMC4696819 26717316

[ref47] Rueden CT, Schindelin J, Hiner MC, DeZonia BE, Walter AE, Arena ET, Eliceiri KW (2017) ImageJ2: ImageJ for the next generation of scientific image data. BMC Bioinform 18:529. 10.1186/s12859-017-1934-z. PMC5708080 29187165

[ref48] Sale PJM (1968) Flushing and leaf growth of cacao under controlled temperature conditions. J Hortic Sci 43:475–489.

[ref49] Sale PJM (1969) Extension growth of cacao under controlled temperature conditions. J Hortic Sci 44:189–193.

[ref50] Sena Gomes AR, Kozlowski TT (1987) Effects of temperature on growth and water relations of cacao (*Theobroma cacao* var. *Comum*) seedlings. Plant Soil 103:3–11.

[ref51] Sena Gomes AR, Kozlowski TT, Reich PB (1987) Some physiological responses of *Theobroma cacao* Var. Catongo seedlings to air humidity. New Phytol 107:591–602.

[ref52] Slot M, Winter K (2016) The effects of rising temperature on the ecophysiology of tropical forest trees. In: Goldstein G, Santiago LS (eds) Tropical tree physiology: adaptations and responses in a changing environment. Springer International Publishing, Switzerland, pp 385–412.

[ref53] Sun P, Mantri N, Lou H, Hu Y, Sun D, Zhu Y, Dong T, Lu H (2012) Effects of elevated CO_2_ and temperature on yield and fruit quality of strawberry (*Fragaria* × *ananassa* Duch.) at two levels of nitrogen application. PloS One 7:e41000. 10.1371/journal.pone.0041000. 22911728 PMC3404062

[ref54] Tserej O, Feeley KJ (2021) Variation in leaf temperatures of tropical and subtropical trees are related to leaf thermoregulatory traits and not geographic distributions. Biotropica 53:868–878.

[ref55] Uddling J, Broberg MC, Feng Z, Pleijel H (2018) Crop quality under rising atmospheric CO_2_. Curr Opin Plant Biol 45:262–267. 29958824 10.1016/j.pbi.2018.06.001

[ref56] Utsunomiya N (1992) Effect of temperature on shoot growth, flowering and fruit growth of purple passionfruit (*Passiflora edulis* Sims var. *edulis*). Sci Hortic 52:63–68.

[ref28] van der Kooi CJ, Reich M, Löw M, De Kok LJ, Tausz M (2016) Growth and yield stimulation under elevated CO_2_ and drought: a meta-analysis on crops. Environ Exp Bot 122:150–157.

[ref57] Woodward FI, Kelly CK (1995) The influence of CO_2_ concentration on stomatal density. New Phytol 131:311–327.

[ref58] Xu Z, Zhou G (2008) Responses of leaf stomatal density to water status and its relationship with photosynthesis in a grass. J Exp Bot 59:3317–3325. 18648104 10.1093/jxb/ern185 PMC2529243

[ref59] Zuidema PA, Heinrich I, Rahman M, Vlam M, Zwartsenberg SA, van der Sleen P (2020) Recent CO_2_ rise has modified the sensitivity of tropical tree growth to rainfall and temperature. Glob Chang Biol 26:4028–4041. 32441438 10.1111/gcb.15092 PMC7317543

