# Supplementary material for: Effects of simulated climate change conditions of increased temperature and [CO2] on the early growth and physiology of the tropical tree crop, Theobroma cacao L
Source: Tree Physiol. 2023 Sep 11;43(12):2050–63. doi: 10.1093/treephys/tpad116 (PMC10714407; doi:10.1093/treephys/tpad116)
Supplement: TP-2023_109_Supplementary_tpad116 [file tp-2023_109_supplementary_tpad116.docx]

**Supporting information**

**
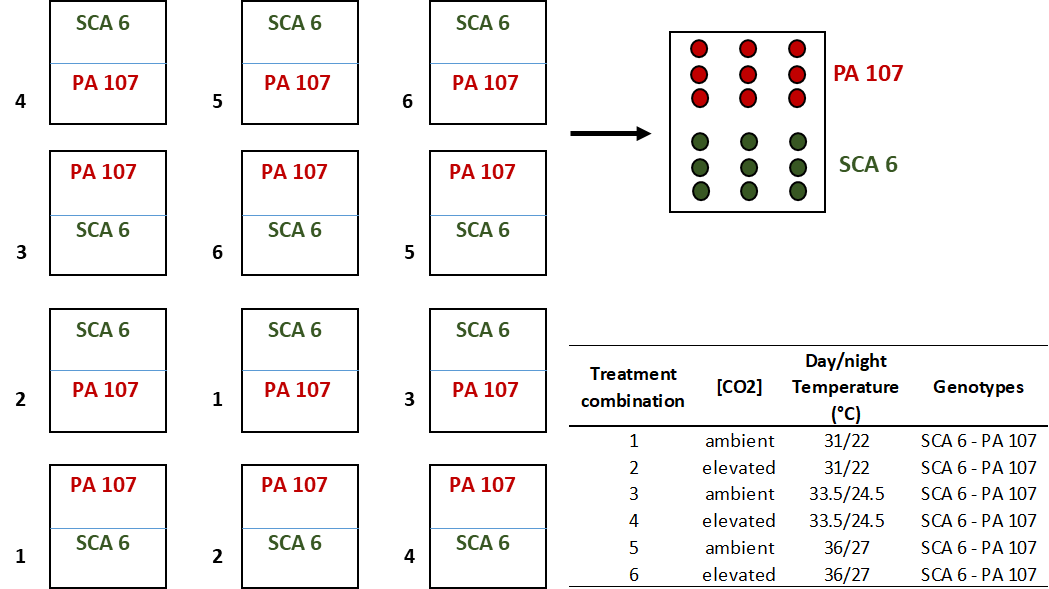
**

**Figure S1** Arrangement of climatic treatment combinations ([CO_2_] * Temperature) and the cacao genotypes across 12 growth cabinets used for the experiment. Each box represents a growth cabinet.

**
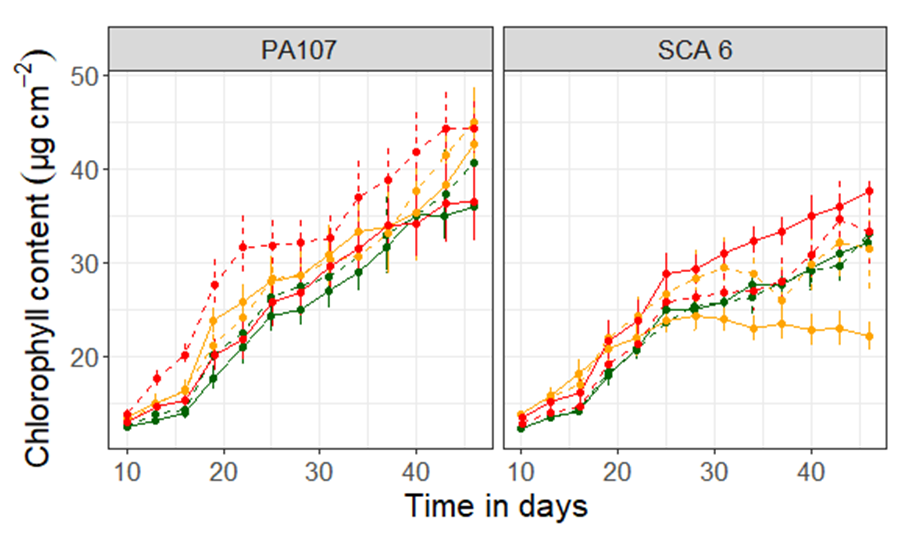
**

**Figure S2** Chlorophyll content measured on two juvenile cacao genotypes grown under two [CO_2_] and three temperatures over 46 days. Error bars show the standard error of the mean (n=6). Temperature treatments are 31/22°C (green), 33.5/24.5°C (orange) and 36/27°C (red). [CO_2_] treatments are ambient (solid lines) and elevated (dashed lines).
